# Greater prefrontal cortical activation is associated with higher balance confidence in older adults

**DOI:** 10.1007/s11357-025-02003-y

**Published:** 2025-11-11

**Authors:** Jin-Young Min, Baek-Yong Choi, Seung-Woo Ryoo, Seok-Yoon Son, Sang-Won Ha, Jihyun Cha, Hanseung Nam, JongKwan Choi, Kyoung-Bok Min

**Affiliations:** 1 Veterans Health Service Medical Center, Veterans Medical Research Institute, Seoul, Republic of Korea; 2 Department of Preventive Medicine, College of Medicine, Seoul National University, 103, Daehak ro, Jongno, Seoul, Republic of Korea; 3 OBELAB Inc., Seoul, Republic of Korea; 4 Integrated Major in Innovative Medical Science, Seoul National University Graduate School, Seoul, Republic of Korea

**Keywords:** Falling, Balance confidence, Prefrontal cortex, fNIRS, Elderly

## Abstract

Fear of falling (FoF) is a prevalent and consequential concern among older adults, often associated with impaired mobility, cognitive decline, and reduced quality of life. Traditionally conceptualized as a psychological response to prior falls, FoF is increasingly recognized as a neurobehavioral phenomenon reflecting dysregulated cognitive-motor integration. In particular, the prefrontal cortex (PFC)—responsible for executive control, attentional regulation, and anticipatory motor planning—has emerged as a key neural substrate underlying FoF. This study investigated the association between PFC activation and balance confidence, a continuous correlate of FoF, in 308 community-dwelling older adults aged ≥ 60 years. Prefrontal oxygenated hemoglobin (HbO) was measured using functional near-infrared spectroscopy (fNIRS) during a verbal fluency task, a standardized cognitive paradigm eliciting PFC engagement without motor interference. Balance confidence was assessed using the validated Korean version of the Activities-specific Balance Confidence (ABC) scale. Subgroup analyses stratified by fall history, age, sex, and educational attainment were conducted to explore heterogeneity by known vulnerability factors. Higher regional HbO levels were significantly associated with higher ABC scores, reflecting greater balance confidence and lower FoF. This association was most pronounced in the right lateral and lower PFC regions (e.g., Right Lateral: β = 1.41, p = 0.0062; Lower Right: β = 1.41, p = 0.0007), and remained robust after adjusting for demographic and clinical covariates. Subgroup analyses revealed stronger associations among individuals with a history of falling, aged ≥ 75 years, women, and those with lower education. For example, in participants with prior falls, Right Hemisphere HbO was strongly correlated with ABC scores (β = 2.06, p = 0.020), suggesting greater cortical recruitment in response to heightened threat perception. We found that greater PFC activation was associated with higher balance confidence in older adults, particularly in those at elevated risk of falling. This relationship may reflect adaptive cortical engagement supporting postural assurance in vulnerable populations.

## Introduction

Fear of falling (FoF) is a prevalent and consequential health concern among older adults. It is commonly defined as an excessive worry about falling or a reduction in balance self-efficacy [30]. Although traditionally conceptualized as a psychological response to prior falls—often labeled as post-fall syndrome—FoF can also develop in individuals with no fall history. Recent global estimates report a pooled prevalence of 49.6%, with substantial heterogeneity across populations [33]. FoF is associated with multiple adverse outcomes, including impaired mobility, gait and balance abnormalities, activity avoidance, and increased risk of physical frailty. These sequelae are further linked to cognitive impairment, social withdrawal, and reduced quality of life [1, 4, 32].

While FoF has historically been regarded as a behavioral or emotional phenomenon, emerging evidence suggests that it may have a neurophysiological basis. In particular, dysregulated cognitive-motor integration has been implicated in older adults with elevated FoF [14, 17, 22]. The prefrontal cortex (PFC)—a hub for executive control, attentional regulation, and anticipatory motor planning—has gained attention as a critical neural substrate in this context [23]. Neuroimaging studies have shown that older adults with high FoF often exhibit increased PFC activation during cognitively or posturally demanding tasks [11, 21]. Holtzer et al. [11] observed exaggerated prefrontal responses and gait slowing during dual-task walking in older adults with high FoF, which were interpreted as signs of neural inefficiency or overload [11]. Salzman et al. [21] also observed prefrontal overactivation in association with fear-induced movement constraints during stair descent [21]. These findings suggest that the PFC plays a pivotal role in monitoring environmental threats and allocating cognitive resources for maintaining balance. However, prior studies have relied on dichotomous classifications of FoF and have employed dual-task paradigms designed to provoke cognitive-motor interference. While informative, such designs may conflate compensatory activation with inefficiency, and may not capture the nuanced, continuous nature of balance confidence. Moreover, these paradigms often lack ecological validity and are limited in their ability to isolate cognitive contributions from motor demands.

To address these gaps, the present study investigated the association between prefrontal cortical activation and balance confidence using a continuous and validated self-report instrument—the Activities-specific Balance Confidence (ABC) scale. Prefrontal oxygenated hemoglobin (HbO) levels were assessed using functional near-infrared spectroscopy (fNIRS) while participants performed a verbal fluency task (VFT), a standardized cognitive paradigm that elicits prefrontal engagement under non-motor conditions. This design allowed us to examine whether greater prefrontal activation corresponds with higher balance confidence, potentially reflecting adaptive cortical support rather than neural inefficiency. In addition, we conducted stratified analyses by fall history, age, sex, and educational attainment—demographic characteristics that are known to influence both FoF risk and cognitive reserve [13, 15, 17]. By identifying subgroup-specific patterns, we aimed to explore whether prefrontal engagement operates differentially across vulnerable populations.

## Methods

### Study population

This study was conducted at the Veterans Health Service Medical Center in Seoul, Republic of Korea, between July and December 2023. Participants were recruited through convenience sampling during routine clinical visits, and eligibility was determined by trained clinicians based on predefined inclusion and exclusion criteria. Inclusion criteria were: (1) age ≥ 60 years; (2) ability to ambulate independently and communicate verbally; (3) absence of major neurological or systemic medical conditions that could influence prefrontal cortical hemodynamics, including cancer, prior cerebral infarction or hemorrhage, Parkinson’s disease, or other progressive neurodegenerative disorders; and (4) absence of physician-diagnosed depression or current use of antidepressants. Individuals with subjective concerns of cognitive decline, but without a formal diagnosis of dementia, were also eligible. Exclusion criteria included: severe mobility impairments precluding safe task performance, inability to complete the verbal fluency task, or technical issues preventing reliable acquisition of fNIRS data.

Based on these criteria, a total of 314 community-dwelling older adults provided informed consent and were enrolled. Of these, 6 participants were subsequently excluded due to incomplete or unreliable fNIRS data (e.g., sensor detachment, drowsiness during testing, or device malfunction). The final analytic sample therefore comprised 308 participants.

### fNIRS data acquisition and preprocessing

**Fig. 1:**
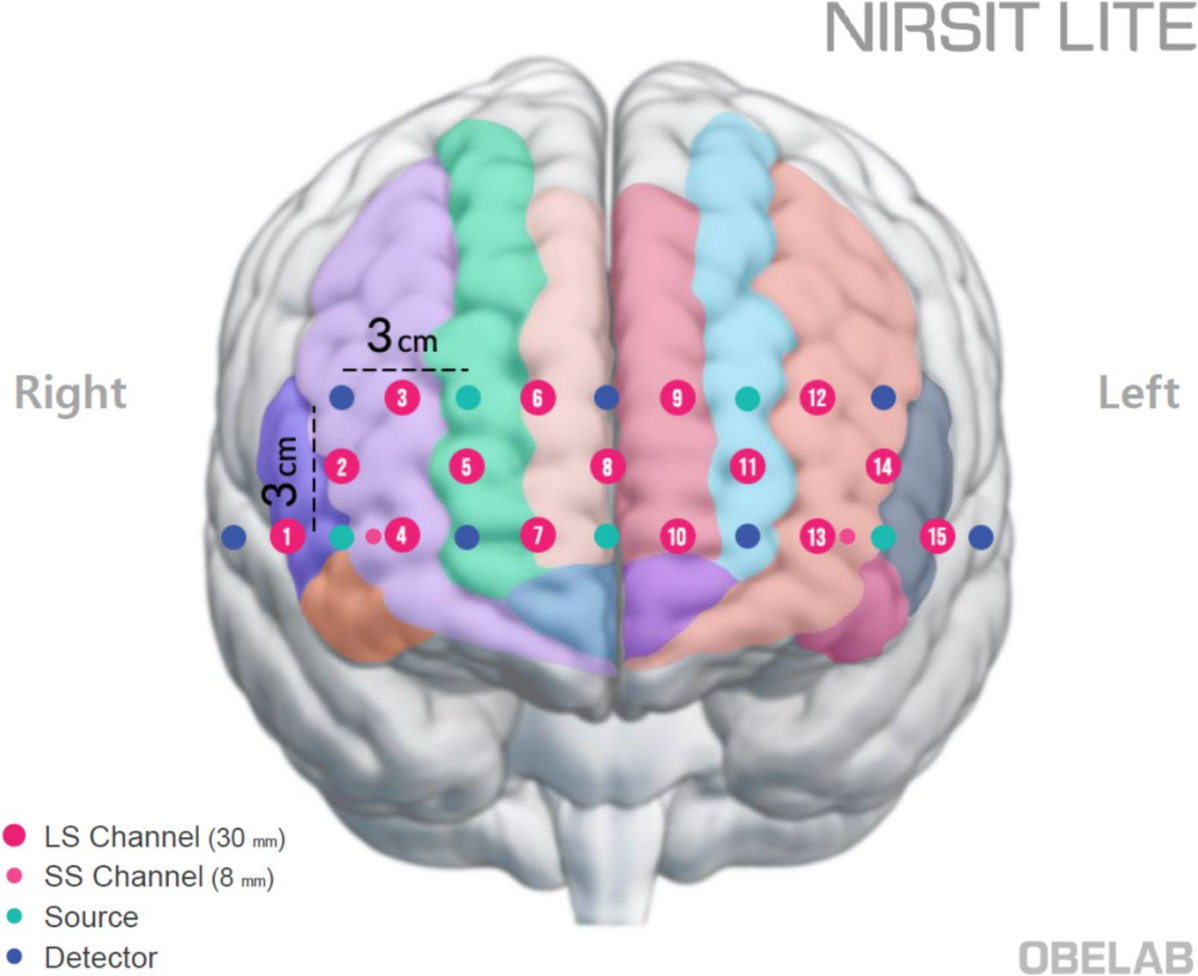
Channel configuration of the fNIRS device. The estimated positions of sources (green dots) and detectors (blue dots) are superimposed on the AAL brain atlas. Each source-detector pair comprises fifteen regular channels (numbered magenta dots) and two short-separation channels (smaller magenta dots). The majority of measurement channels were positioned over the anterior prefrontal cortex, with some (channels 1 and 15) located over the inferior frontal gyri

A portable continuous-wave fNIRS device (NIRSIT Lite Adult; OBELAB, Inc., Seoul, Korea) was used to measure hemodynamic responses in the PFC during the task. The system included 15 channels with a 3 cm source-detector distance and used dual wavelengths (780 and 850 nm) at a sampling rate of 8.138 Hz. It was also equipped with two short-separation channels, one in each hemisphere, with a source-detector distance of 0.8 cm. The device primarily targeted the anterior PFC (BA10), approximately covering adjacent regions including the dorsolateral and ventrolateral PFC. Figure 1 illustrates the configuration of the channels and their estimated positions on the automated anatomical labeling (AAL) atlas [20].

During fNIRS data acquisition, participants performed a verbal fluency task using a tablet PC that presented the instructions and stimuli. The task consisted of two cycles, each comprising three sequential phases. In each cycle, participants first engaged in a 30-s resting period, during which they were instructed to fixate on a central cross displayed on the screen. This was followed by a 30-s control condition, where they repeatedly uttered vowels (“a, e, i, o, u”) aloud. Finally, in the 30-s task condition, participants were presented with an initial Korean consonant (‘Giyuk’ in the first cycle and ‘Siot’ in the second cycle) and asked to generate and verbally produce as many words as possible beginning with the presented consonant.

The preprocessing steps for the fNIRS data were as follows. First, channels with low signal quality due to saturation or severe motion artifacts were excluded from the analysis [35]. Specifically, the raw light intensity signal in each channel was examined and removed if it met any of the following criteria: (1) contained signs of saturation (more than five negative values in a row or the same consecutive value persisting for more than 5% of the entire time series); (2) showed weak net signal (median intensity lower than 30 A.U.); (3) exhibited signs of noise (a coefficient of variation greater than 7.5% in more than 10% of 5-s intervals [2]; or (4) demonstrated severe negative correlation between HbO and deoxyhemoglobin (HbR) concentrations [28]. Second, the raw signals were converted to optical density and corrected for motion artifacts using the Temporal Derivative Distribution Repair (TDDR) algorithm [8]. Third, the corrected optical density values were converted to changes in hemoglobin concentration via the modified Beer-Lambert law [6, 7]. For this conversion, the molar extinction coefficients reported by Zhao et al. [36] were applied, with no differential pathlength factor, resulting in hemoglobin concentration units of mM·mm [36]. Finally, data were bandpass filtered between 0.005 and 0.1 Hz to remove frequencies of no interest such as cardiac pulsation and slow drifts. Overall, less than 1% of the total observations were rejected during preprocessing, and these were replaced by the mean of nearby channels. All preprocessing and subsequent statistical analyses described in the next section were performed using NIRSIT Quest software (version 1.0.0; OBELAB, Inc., Seoul, Korea).

### fNIRS data analysis

To quantify and summarize brain activation during the verbal fluency task, a general linear model (GLM) was applied to the hemoglobin signals. Beta coefficients were estimated using the Ordinary Least Squares (OLS) method [9]. The design matrix included main regressors modeling the task condition (verbal fluency) and the control condition (vowel utterance), along with their temporal and dispersion derivatives to account for variability in response timing. To further improve model fit and reduce confounding influences, nuisance regressors were incorporated. Any residual motion artifacts were addressed by including three head movement angle estimates (rotation in x, y, and z directions) derived from the inertial measurement unit (IMU) sensors [18]. Additionally, signals from short-separation channels were averaged and included as nuisance regressors to remove superficial physiological noise [10, 31]. The model was independently fitted to the concentration changes in.

**Fig. 2:**
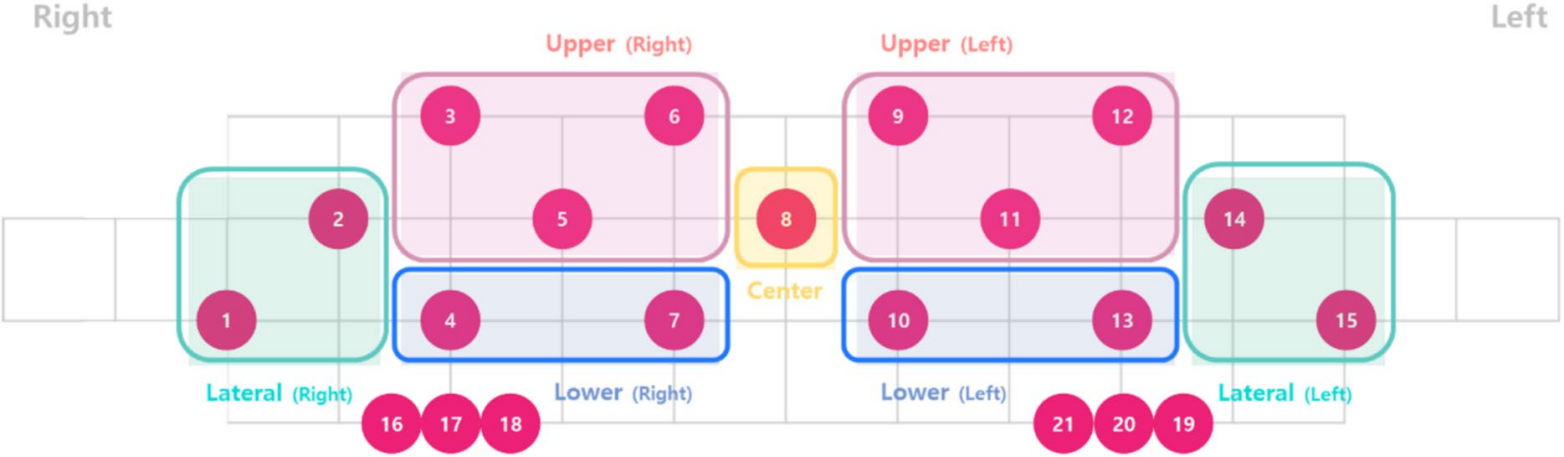
Pre-defined regions of interest based on channel clustering. Activation in each channel was summarized across channel groups defined by hierarchical clustering of functional connectivity patterns derived from an independent dataset (excerpted with permission from OBELAB Inc. [16])

HbO and HbR. Task-related prefrontal activation for each participant was defined by combining the parameter estimates using a contrast vector of [1–1], representing Task versus Control. Channel-wise contrasted beta estimates were computed and summarized at the regional level by averaging estimates across predefined regions of interest (Fig. 2). For details regarding ROI definitions, see OBELAB Inc. ([16]) (Obelab Inc, [16]). We primarily focused on HbO results, as previous studies have demonstrated that HbO generally provides a higher contrast-to-noise ratio compared to HbR [26].

### Assessment of FoF

Fear of falling was measured using the Korean version of the ABC scale, a validated instrument that assesses individuals’ confidence in performing 16 common daily activities without losing balance. These activities encompass a range of scenarios such as walking indoors and outdoors, reaching overhead, navigating stairs, and using escalators. Participants rated their confidence for each item on a scale from 0 (no confidence) to 100 (complete confidence), and the final ABC score was calculated as the average of the 16 items [19]. Higher scores indicate greater balance confidence and therefore lower levels of FoF.

The Korean version of the ABC scale has demonstrated strong psychometric properties. Its translation fidelity was confirmed via a forward–backward translation procedure, and previous studies have reported high internal consistency (Cronbach’s α = 0.96) and concurrent validity with other fall-related measures such as the Falls Efficacy Scale (Spearman’s ρ = 0.78, p < 0.01), supporting its use in Korean older adult populations [12].

### Variables of interest

Demographic and health-related covariates were included to contextualize the relationship between cerebral hemodynamics and balance confidence. Age was categorized into six groups (60–64, 65–69, 70–74, 75–79, 80–84, and ≥ 85 years). Other variables included sex (male or female), marital status (married, widowed, divorced, or never married), and educational attainment (less than high school vs. high school or more). Smoking status was self-reported as current, former, or never smoker. Participants also indicated whether they had ever experienced a fall (yes or no). Clinical comorbidities were identified based on self-reported physician diagnoses of hypertension, dyslipidemia, and diabetes mellitus. Additionally, the number of medications currently taken was recorded and categorized as 0, 1, or ≥ 2. Global cognitive function was evaluated using the Mini-Mental State Examination (MMSE), a widely used screening tool for cognitive impairment in older adults. The MMSE assesses orientation, attention, memory, language, and visuospatial skills, with scores ranging from 0 to 30, where higher scores reflect better cognitive performance. This measure was included as a covariate to account for potential confounding, given that cognitive status may influence both FoF and PFC activation during the verbal fluency task.

### Statistical analysis

We hypothesized that prefrontal hemodynamic activity—indexed by HbO measured via fNIRS—would be positively associated with balance confidence in older adults. To test this hypothesis, we performed both unadjusted and multivariable linear regression analyses, using HbO concentrations in predefined frontal cortical regions as independent variables and ABC scores as the continuous dependent outcome. The fully adjusted model controlled for potential confounders, including age, sex, marital status, education level, smoking status, fall experience, chronic disease history (hypertension, dyslipidemia, diabetes), medication count, and cognitive function. Beta coefficients and standard errors (SE) were estimated to quantify the association between HbO levels and ABC scores.

In addition, we conducted subgroup analyses stratified by fall history, age group, sex, and educational attainment to examine whether the association between PFC activation and balance confidence varied across key sociodemographic and clinical characteristics. For each subgroup, both unadjusted and adjusted linear regression models were estimated to assess the consistency and robustness of the associations. These analyses aimed to identify potential effect modification by vulnerability factors known to influence both FoF and cortical activation patterns. To enhance visualization of cortical activation contrasts, we additionally illustrated the most extreme subgroups, defined as participants with high ABC scores (≥ 80) and those with low ABC scores (≤ 40). This stratification was applied solely for graphical presentation and was not used in the main statistical analyses.

All statistical analyses were performed using SAS version 9.2 (SAS Institute, Cary, NC, USA), and statistical significance was defined as a two-tailed p-value < 0.05.

## Results

As shown in Table 1, the study included 308 older adults. The majority were in their 70 s (69.81%) and slightly more than half were male (52.27%). Most participants were married (78.25%), followed by widowed (16.23%), divorced (4.87%), and never married (0.65%). Educational attainment was generally low, with 71.8% of participants having completed high school or less. Approximately 22.08% of participants reported a history of falling. Additionally, a large proportion had hypertension (60.71%) and were taking more than two medications (85.39%). The mean MMSE score of participants was 27.1 (SD = 2.2), indicating generally preserved global cognitive function within the study sample. 

**Table 1:** Characteristics of study population (*n* = 308)

Variables	n	(%)
Age (year)		
60–64	8	(2.6)
65–69	34	(11.04)
70–74	100	(32.47)
75–79	115	(37.34)
80–84	45	(14.61)
≥ 85	6	(1.95)
Sex		
Female	147	(47.73)
Male	161	(52.27)
Marital status		
Married	241	(78.25)
Widowed	50	(16.23)
Divorced	15	(4.87)
Never married	2	(0.65)
Educational attainment		
Less than a high school diploma	221	(71.75)
More than a high school diploma	87	(28.25)
Cigarette smoking		
Current smoker	13	(4.22)
Past smoker	108	(35.06)
Never smoked	187	(60.71)
Falling experience		
Yes	68	(22.08)
No	240	(77.92)
History of disease		
Hypertension	187	(60.71)
Dyslipidemia	84	(27.27)
Diabetes	28	(9.09)
Number of medications		
0	17	(5.52)
1	28	(9.09)
≤ 2	263	(85.39)
MMSE score, mean (SD)	27.08	(2.18)

**Fig. 3:**
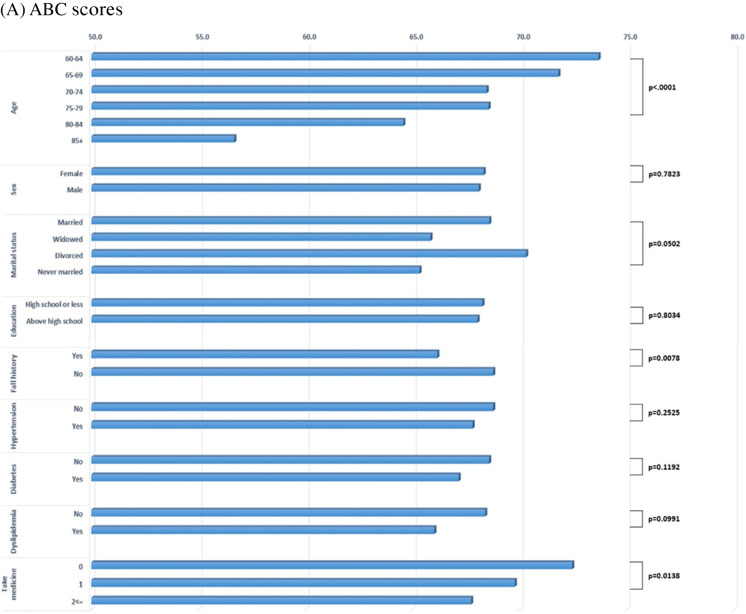

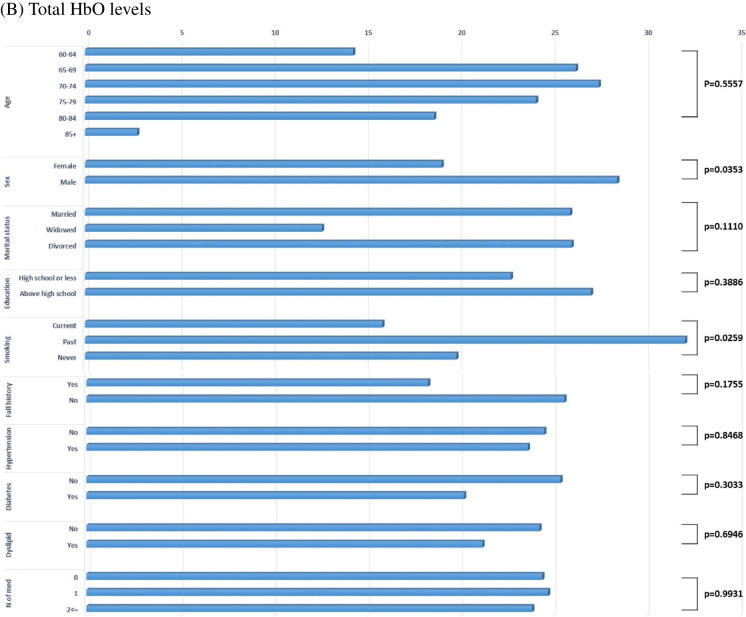
Mean distribution of (**A**) ABC scores and (**B**) total HbO levels across participant characteristics. The blue-bar displays the mean ABC scores (blue bars) or total HbO levels by demographic and clinical variables. P-values indicate the statistical significance of between-group differences in FoF scores or total HbO levels. HTN, hypertension; Dyslipid, dyslipidemia; No. of med, number of medications

Figure 3 illustrates the distribution of (A) ABC scores and (B) total HbO levels across participant characteristics. Higher ABC scores indicate greater balance confidence and therefore lower levels of FoF. ABC scores declined significantly with increasing age (p < 0.0001), and individuals with a history of falling demonstrated significantly lower ABC scores than those without (p = 0.0078). Participants taking more than two medications also exhibited the lowest ABC scores (p = 0.0138). No significant differences in ABC scores were observed by sex, marital status, educational attainment, smoking status, or presence of hypertension, dyslipidemia, or diabetes. Regarding cerebral hemodynamics, mean total HbO levels were significantly higher in males than females (p = 0.0353). HbO levels were significantly higher among married participants compared with unmarried individuals (p = 0.011), and among never-smokers compared with former smokers (p = 0.026).

Table 2 presents the results of linear regression analyses examining the association between ABC scores and regional HbO levels measured by fNIRS. In nearly all brain regions—except the central region—higher HbO levels were significantly associated with higher ABC scores, indicating greater balance confidence and lower FoF. These associations remained robust after adjusting for potential confounders, including age, sex, marital status, education, smoking, fall history, chronic disease history, number of medications, and MMSE score. The strongest associations were observed in the right hemisphere, particularly in the Right Lateral (β = 1.41; p = 0.0062), Lower Right (β = 1.41; p = 0.0007), and Right Hemisphere overall (β = 1.32; p = 0.0008) regions. Other significant associations were found in the Upper Right (β = 1.13; p = 0.0093), Upper Left (β = 1.05; p = 0.0078), Left Lateral (β = 1.21; p = 0.0362), Lower Left (β = 0.94; p = 0.0264), Left Hemisphere overall (β = 1.07; p = 0.0047), and the Total PFC region (β = 1.08; p = 0.0008). The central region showed no significant association (β = 0.44; p = 0.4247). 

**Table 2:** Association between ABC scores and regional HbO levels measured by fNIRS

Brain regions	Unadjusted model			Adjusted model^*^		
	Beta	(SE)	*p*-value	Beta	(SE)	*p*-value
Upper Right	1.19	(0.38)	0.0018	**1.13**	**(0.43)**	**0.0093**
Upper Left	1.15	(0.34)	0.0009	**1.05**	**(0.39)**	**0.0078**
Right Lateral	1.57	(0.45)	0.0005	**1.41**	**(0.51)**	**0.0062**
Left Lateral	1.46	(0.51)	0.0043	**1.21**	**(0.58)**	**0.0362**
Lower Right	1.34	(0.36)	0.0003	**1.41**	**(0.41)**	**0.0007**
Lower Left	1.00	(0.37)	0.0074	**0.94**	**(0.42)**	**0.0264**
Center	0.41	(0.49)	0.4025	0.44	(0.55)	0.4247
Total	1.16	(0.31)	0.0002	**1.08**	**(0.35)**	**0.0022**
Right	1.37	(0.34)	<.0001	**1.32**	**(0.39)**	**0.0008**
Left	1.21	(0.33)	0.0003	**1.07**	**(0.37)**	**0.0047**

^*^Adjusted by age, sex, marital status, education, smoking, falling experience, history of disease, number of medications, and MMSE score

**Fig. 4:**
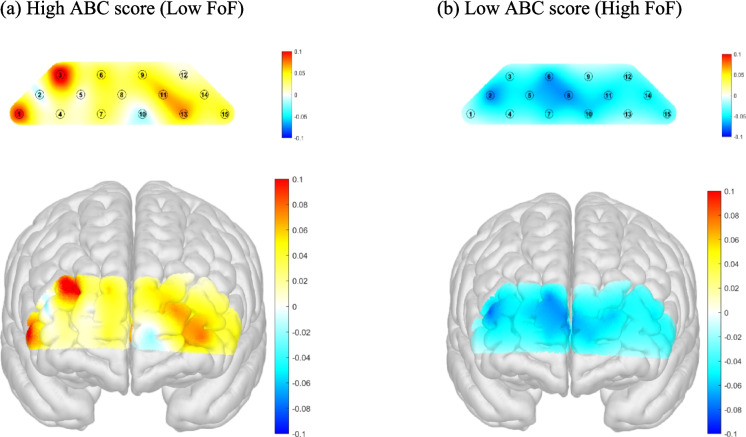
Comparison of prefrontal cortical activation (HbO) during verbal fluency task between high and low balance confidence groups. High balance confidence was defined as an ABC score ≥ 80, and low balance confidence as an ABC score ≤ 40. Topographic and 3D cortical maps illustrate the distribution of HbO levels in the prefrontal cortex among participants with high ABC scores (Low FoF; panel **a**) and low ABC scores (High FoF; panel **b**). Warmer colors (yellow to red) indicate increased HbO concentration, reflecting greater cortical activation, whereas cooler colors (blue) indicate decreased HbO levels, reflecting lower activation

Figure 4 illustrates the spatial distribution of prefrontal cortical activation, indexed by HbO levels, during a verbal fluency task in older adults stratified by balance confidence level. Panel (a) shows the high ABC score group (low fear of falling), which demonstrated widespread increases in HbO, particularly in the lateral and inferior prefrontal regions, with greater activation evident in the right hemisphere. In contrast, panel (b) depicts the low ABC score group (high fear of falling), which exhibited overall reductions in HbO across prefrontal areas, as indicated by predominance of blue-shaded regions.

Table 3 presents stratified linear regression analyses examining the association between ABC scores and regional HbO levels by history of falling. Among participants with a prior fall, higher HbO levels across most prefrontal regions were significantly associated with higher ABC scores, indicating greater balance confidence and reduced FoF. The strongest associations were observed in the Lower Right (β = 2.15; p = 0.0024), Right Hemisphere (β = 2.06; p = 0.0021), and Right Lateral (β = 2.10; p = 0.0165) regions. Other regions showing significant associations included the Upper Right (β = 1.94; p = 0.0084), Upper Left (β = 1.68; p = 0.0119), Lower Left (β = 1.39; p = 0.0211), Left Hemisphere (β = 1.53; p = 0.0085), and the Total region (β = 1.75; p = 0.0028). In contrast, among those without a prior fall, associations were generally weaker and more limited in statistical significance. Significant associations were detected in the Right Lateral (β = 1.53; p = 0.0225), Lower Right (β = 1.39; p = 0.0106), Right Hemisphere (β = 1.34; p = 0.0089), and Total region (β = 0.98; p = 0.0344). Other regions showed marginal or non-significant associations.

**Table 3:** Stratified associations between ABC scores and regional HbO levels by fall history

Brain regions	With falling experience			Without falling experience		
	Beta^*^	(SE)	p-value	Beta^*^	(SE)	p-value
Upper Right	**1.94**	**(0.71)**	**0.0084**	1.10	(0.57)	0.0541
Upper Left	**1.68**	**(0.64)**	**0.0119**	1.01	(0.52)	0.0516
Right Lateral	**2.10**	**(0.85)**	**0.0165**	**1.53**	**(0.67)**	**0.0225**
Left Lateral	1.51	(0.91)	0.1040	1.04	(0.74)	0.1603
Lower Right	**2.15**	**(0.67)**	**0.0024**	**1.39**	**(0.54)**	**0.0106**
Lower Left	**1.39**	**(0.58)**	**0.0211**	0.75	(0.57)	0.1935
Center	1.45	(1.09)	0.1910	0.05	(0.70)	0.9407
Total	**1.75**	**(0.56)**	**0.0028**	**0.98**	**(0.46)**	**0.0344**
Right	**2.06**	**(0.63)**	**0.0020**	**1.34**	**(0.51)**	**0.0089**
Left	**1.53**	**(0.56)**	**0.0085**	0.93	(0.50)	0.0612

^*^Adjusted by age, sex, marital status, education, smoking, falling experience, history of disease, number of medications, and MMSE score.

Table 4 presents stratified linear regression analyses evaluating the association between ABC scores and regional HbO levels across demographic subgroups, including age, sex, and educational attainment. All models were adjusted for relevant covariates, and the analyses aimed to identify subgroup-specific differences in the association between balance confidence and prefrontal activation. Age-stratified analyses showed that the associations between HbO levels and ABC scores were generally stronger and more statistically significant among older adults (≥ 75 years) compared to those under 75. Significant associations in the ≥ 75 group were found in the Upper Right (β = 1.42; p = 0.0215), Upper Left (β = 1.28; p = 0.0174), Right Lateral (β = 1.75; p = 0.0161), and Left Hemisphere (β = 1.14; p = 0.0231). In contrast, while some brain regions showed positive trends in the < 75 group, none reached statistical significance, suggesting that prefrontal activation may play a more prominent role in promoting balance confidence among the older-old population. Sex-stratified analyses indicated that the positive association between HbO and ABC scores was more pronounced in females. Females demonstrated significant associations in several regions, including Upper Right (β = 1.28; p = 0.0168), Right Lateral (β = 2.06; p = 0.0065), and Right Hemisphere (β = 1.63; p = 0.0014). In contrast, males showed generally weaker and non-significant associations across regions, suggesting possible sex differences in the neural mechanisms underlying balance confidence. Education-stratified analyses showed that individuals with lower educational attainment (high school or less) exhibited stronger associations between HbO and ABC scores. Notable associations were found in the Right Lateral (β = 1.47; p = 0.0071), Left Lateral (β = 1.32; p = 0.0463), Lower Right (β = 1.51; p = 0.0009), and Total region (β = 1.36; p = 0.0018). In contrast, among those with higher education, most associations were attenuated and did not reach statistically significance, except for Upper Right (β = 2.11; p = 0.0257) and Upper Left (β = 2.03; p = 0.0356).

**Table 4:**
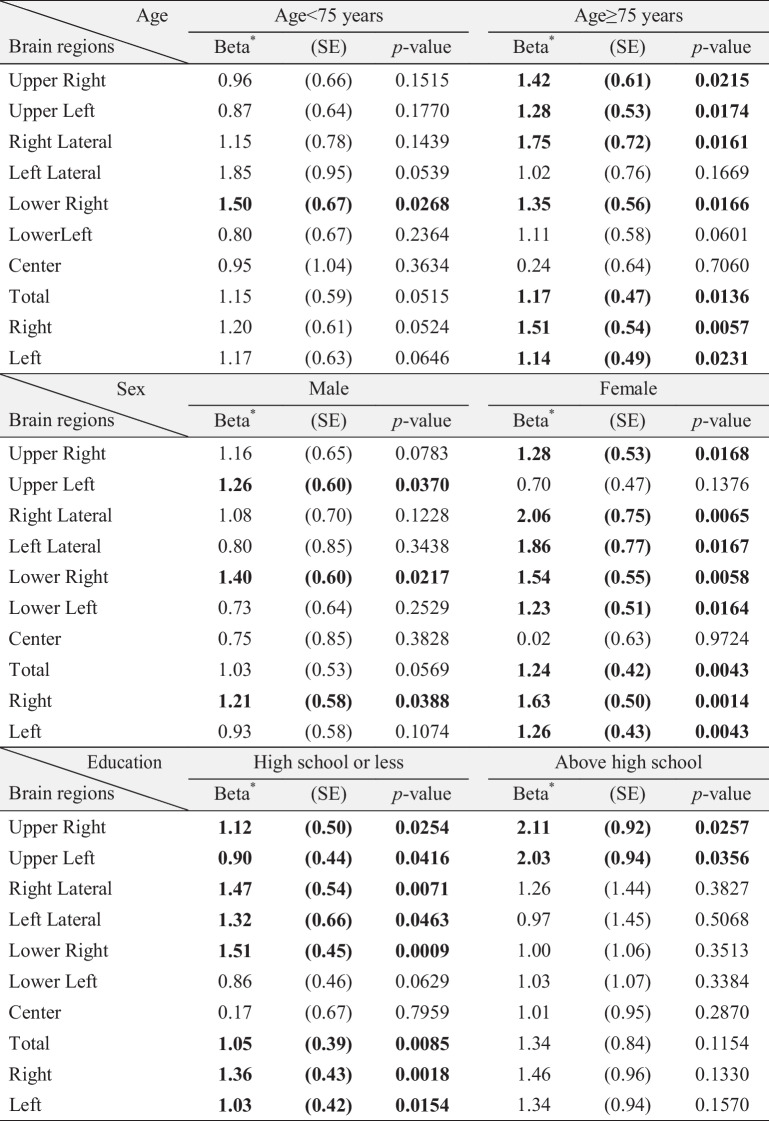
Stratified associations between ABC scores and regional HbO levels by age, sex, and educational attainment

*The Beta coefficients represent the estimated associations between ABC scores and HbO levels. All models were adjusted for age, sex, marital status, educational attainment, smoking status, history of chronic diseases, number of medications, and MMSE score, except for the stratifying variable in each analysis (e.g., sex or educational attainment was not included as a covariate in the corresponding stratified models)

## Discussion

Our findings demonstrate that prefrontal cortical activation—indexed by HbO levels during a verbal fluency task—is positively associated with balance confidence, as measured by the ABC scale. Specifically, individuals with higher balance confidence exhibited significantly greater prefrontal activation, suggesting that increased cortical engagement may support postural confidence and reduce fear of falling. Importantly, these associations remained robust after adjustment for cognitive status, as measured by MMSE scores, indicating that the observed relationship is not explained by baseline cognitive function.

These results partially diverge from previous studies, which have predominantly interpreted elevated prefrontal activity in individuals with high FoF as a marker of neural inefficiency or cognitive-motor interference. Notably, prior research has often employed dichotomized FoF classifications and dual-task gait paradigms designed to provoke cognitive-motor conflict [11, 25]. For instance, Holtzer et al. [11] observed that older adults with high FoF exhibited increased prefrontal HbO during dual-task walking, along with slower gait and prolonged cortical activation—findings interpreted as reflecting inefficient or overloaded neural systems [11]. Stojan et al. [25] also reported that elevated prefrontal and parietal activation during cognitively demanding gait tasks predicted poorer behavioral outcomes, reinforcing the inefficiency hypothesis [25].

In contrast, our study employed a continuous measure of balance confidence and a single cognitive task under controlled, non-motor conditions. This methodological approach allowed for the detection of graded associations between prefrontal activation and balance confidence, independent of motor interference. Notably, increased HbO levels—particularly in the lateral and inferior subregions of the prefrontal cortex—were significantly associated with higher ABC scores, indicating greater balance confidence and lower fear of falling. These findings suggest that heightened prefrontal engagement may reflect an adaptive, compensatory mechanism that supports postural confidence, even under modest cognitive demands. Taken together, our results offer novel insights into the cortical correlates of FoF and highlight the utility of continuous, task-independent paradigms in detecting subclinical variations in neural function associated with balance regulation.

Although direct empirical evidence linking balance confidence with increased prefrontal activation remains limited, our findings align with previous studies suggesting that cognitive resources—particularly prefrontal engagement—play a compensatory role in supporting postural control. A study of Xu et al. [34] demonstrated that individuals with mild cognitive impairment exhibited heightened prefrontal activation under dual-task balance conditions, likely reflecting adaptive cortical recruitment to maintain stability [34]. Teo et al. [29] observed increased dorsolateral PFC activation in older adults subjected to sensory perturbations during quiet standing, which was positively associated with improved postural performance [29]. Chen et al. [5] further reported that successful obstacle negotiation during dual-task walking was facilitated by enhanced PFC activation and functional connectivity, reinforcing the role of the prefrontal cortex in dynamic balance regulation [5]. Collectively, these studies support the interpretation that prefrontal recruitment may serve as an adaptive mechanism for sustaining postural confidence in cognitively or sensorimotorically demanding contexts.

While the underlying neurobiological mechanisms remain incompletely understood, several plausible pathways may explain our findings. First, increased PFC activation may reflect top-down modulation of postural control systems through anticipatory and executive processes. Takakusaki’s [27] neuroanatomical model suggests that the prefrontal cortex contributes to balance regulation by generating internal representations of body orientation, planning goal-directed motor sequences, and exerting control over motor output via its projections to the premotor cortex, supplementary motor area, basal ganglia, and brainstem locomotor centers [27]. Such cortical involvement likely supports postural stability even under low motor demands, particularly when cognitive vigilance is required. Second, individuals with preserved or enhanced cognitive reserve may exhibit more efficient recruitment of prefrontal networks to support balance confidence. According to Stern’s [24] cognitive reserve framework, higher reserve enables flexible neural resource allocation to optimize behavioral outcomes [24]. In our study, increased prefrontal activation may thus reflect an active, efficient response rather than compensatory overload—particularly among those with greater postural confidence. Third, adaptive neuroplasticity and intact neurovascular coupling in older adults may facilitate this engagement. As Cabeza et al. [3] proposed, successful cognitive aging is characterized by the compensatory recruitment of additional neural systems to maintain functional performance as core networks decline [3]. Our findings suggest that this compensation may extend to the domain of balance confidence, allowing older adults to proactively engage prefrontal circuits to reinforce postural control and reduce FoF.

Our stratified analyses further revealed that the positive association between prefrontal HbO levels and balance confidence was most pronounced among individuals with a history of falls, those aged ≥ 75 years, women, and those with lower educational attainment. These subgroup effects may reflect shared compensatory mechanisms, whereby individuals at elevated risk of falling—due to prior trauma, age-related decline, or limited cognitive reserve—engage prefrontal resources more intensively to maintain postural stability and self-assurance. Collectively, these findings challenge the conventional view that increased prefrontal activation solely reflects neural inefficiency. Instead, they support a more nuanced and context-sensitive interpretation: that elevated prefrontal engagement may serve an adaptive cortical function, particularly in vulnerable populations.

Several limitations of this study warrant consideration. First, disease severity was not directly assessed beyond comorbidity and medication counts. We operationalized polypharmacy as a categorical indicator (0, 1, or ≥ 2 medications), which provides a pragmatic approximation of health burden but does not fully capture the complexity of pharmacotherapy. Specifically, we did not distinguish between fixed-dose combinations and separate agents, nor did we account for dosage variations or apply standardized metrics such as ATC/DDD. Consequently, residual confounding related to multimorbidity, therapeutic duplication, and dose intensity may remain. Because polypharmacy was used only as an adjustment covariate rather than as a primary exposure, such misclassification is more likely to attenuate confounding control than to create spurious associations. Accordingly, our estimates should be interpreted conservatively, and future studies incorporating ingredient-level coding, dose normalization, and appropriateness frameworks (e.g., Beers, STOPP/START criteria) will be essential for more precise adjustment. Second, the cross-sectional design precludes causal inference, limiting our ability to determine whether prefrontal activation facilitates higher balance confidence or instead represents a compensatory response to diminished confidence or FoF. Longitudinal or experimental designs will be essential to clarify the directionality of this relationship. Third, while the verbal fluency task provided a controlled cognitive stimulus without motor interference, it may not fully capture the complex interactions between cognitive and motor demands encountered in daily life. Future studies incorporating dual-task or ecologically valid tasks may yield deeper insights into real-world mechanisms. Fourth, although HbO is widely accepted as a proxy for cortical activation in fNIRS studies, it may be influenced by extracerebral hemodynamic artifacts. Despite employing standardized preprocessing and probe localization procedures, integration with multimodal neuroimaging techniques (e.g., EEG-fNIRS or fMRI) could enhance spatial specificity and interpretability. Fifth, we did not collect information on diet, dietary supplementation, physical activity, or sleep quality—all of which are known to influence cortical hemodynamics and fall-related confidence. The omission of these factors may have introduced residual confounding and should be addressed in future studies. Moreover, dietary supplementation was not considered when assessing polypharmacy, which further limits interpretation of medication burden in this population. Finally, the sample size within certain stratified subgroups (e.g., individuals with low educational attainment or a history of falls) was relatively limited, potentially constraining statistical power and the generalizability of subgroup-specific findings.In conclusion, our findings indicate that prefrontal cortical activation is positively associated with balance confidence in older adults, particularly among those at elevated risk of falling. This association may reflect an adaptive neural response supporting postural control under conditions of perceived threat. By providing neurophysiological insight into the cognitive mechanisms underlying fear of falling, these results underscore the potential relevance of prefrontal function as both a marker of fall-related vulnerability and a modifiable target for intervention. Continued research using longitudinal and ecologically grounded approaches is warranted to clarify causal pathways and inform strategies that support mobility and confidence in aging populations.

## Data Availability

The dataset generated and analyzed in the current study is available from the corresponding author on reasonable request.

## References

[1] Auais M, Alvarado B, Guerra R, Curcio C, Freeman EE, Ylli A, et al. Fear of falling and its association with life-space mobility of older adults: a cross-sectional analysis using data from five international sites. Age Ageing. 2017;46:459–65. 28043980 10.1093/ageing/afw239 PMC5405754

[2] Bonilauri A, Sangiuliano Intra F, Baselli G, Baglio F. Assessment of fNIRS signal processing pipelines: towards clinical applications. Appl Sci. 2021;1:316.

[3] Cabeza R, Albert M, Belleville S, Craik FIM, Duarte A, Grady CL, et al. Maintenance, reserve and compensation: the cognitive neuroscience of healthy ageing. Nat Rev Neurosci. 2018;19:701–10. 30305711 10.1038/s41583-018-0068-2 PMC6472256

[4] Chang HT, Chen HC, Chou P. Factors associated with fear of falling among community-dwelling older adults in the Shih-pai study in Taiwan. PLoS One. 2016;11:e0150612. 26933882 10.1371/journal.pone.0150612 PMC4775068

[5] Chen Y, Cao Z, Mao M, Sun W, Song Q, Mao D. Increased cortical activation and enhanced functional connectivity in the prefrontal cortex ensure dynamic postural balance during dual-task obstacle negotiation in the older adults: a fNIRS study. Brain Cogn. 2022;163:105904. 36063567 10.1016/j.bandc.2022.105904

[6] Cope M, Delpy DT. System for long-term measurement of cerebral blood and tissue oxygenation on newborn infants by near infra-red transillumination. Med Biol Eng Comput. 1988;26:289–94. 2855531 10.1007/BF02447083

[7] Delpy DT, Cope M, van der Zee P, Arridge S, Wray S, Wyatt J. Estimation of optical pathlength through tissue from direct time of flight measurement. Phys Med Biol. 1988;33:1433–42. 3237772 10.1088/0031-9155/33/12/008

[8] Fishburn FA, Ludlum RS, Vaidya CJ, Medvedev AV. Temporal derivative distribution repair (TDDR): a motion correction method for fNIRS. Neuroimage. 2019;184:171–9. 30217544 10.1016/j.neuroimage.2018.09.025 PMC6230489

[9] Friston KJ, Penny WD, Ashburner J, Kiebel SJ, Nichols TE. Statistical parametric mapping: the analysis of functional brain images. Academic Press; 2006.

[10] Gagnon L, Perdue K, Greve DN, Goldenholz D, Kaskhedikar G, Boas DA. Improved recovery of the hemodynamic response in diffuse optical imaging using short optode separations and state-space modeling. Neuroimage. 2011;56:1362–71. 21385616 10.1016/j.neuroimage.2011.03.001 PMC3085546

[11] Holtzer R, Kraut R, Izzetoglu M, Ye K. The effect of fear of falling on prefrontal cortex activation and efficiency during walking in older adults. Geroscience. 2019;41:89–100. 30737727 10.1007/s11357-019-00056-4 PMC6423209

[12] Jang SN, Cho SI, Oh SW, Lee ES, Baik HW, Lee HJ. The validity and reliability of Korean Fall Efficacy Scale (FES) and Activities-specific Balance Confidence Scale (ABC). J Korean Geriatr Soc. 2003;7:255–68.

[13] Kim T, Choi SD, Xiong S. Epidemiology of fall and its socioeconomic risk factors in community-dwelling Korean elderly. PLoS One. 2020;15:e0234787. 32559206 10.1371/journal.pone.0234787 PMC7304594

[14] Kraut R, Holtzer R. Recurrent but not single report of fear of falling predicts cognitive decline in community-residing older adults. Aging Ment Health. 2022;26:100–6. 33938782 10.1080/13607863.2021.1916878

[15] Lee S, Oh E, Hong GS. Comparison of Factors Associated with Fear of Falling between Older Adults with and without a Fall History. Int J Environ Res Public Health, 2018:15:982 10.3390/ijerph15050982 PMC5982021 29757960

[16] Obelab Inc. NIRSIT LITE Channel Information, Seoul, Korea https://www.obelab.com/info/notice.php. OBELAB Inc. 2022. Accessed 29 Sept 2025.

[17] Peeters G, Bennett M, Donoghue OA, Kennelly S, Kenny RA. Understanding the aetiology of fear of falling from the perspective of a fear-avoidance model - a narrative review. Clin Psychol Rev. 2020;79:101862. 32442854 10.1016/j.cpr.2020.101862

[18] Penny WD, Friston KJ, Ashburner J, Kiebel SJ, Nichols TE. Statistical parametric mapping: the analysis of functional brain images. Academic Press; 2011.

[19] Powell LE, Myers AM. The activities-specific balance confidence (ABC) scale. J Gerontol A Biol Sci Med Sci. 1995;50a:M28-34. 7814786 10.1093/gerona/50a.1.m28

[20] Rolls ET, Huang CC, Lin CP, Feng J, Joliot M. Automated anatomical labelling atlas 3. Neuroimage. 2020;206:116189. 31521825 10.1016/j.neuroimage.2019.116189

[21] Salzman T, Aboualmagd A, Badawi H, Tobón-Vallejo D, Kim H, Dahroug L, et al. Prefrontal cortex involvement during dual-task stair climbing in healthy older adults: an fNIRS study. Brain Sci. 2021. 10.3390/brainsci11010071. 33430358 10.3390/brainsci11010071 PMC7825747

[22] Son SY, Kim CY, Choi BY, Ryoo SW, Oh KH, Min JY, et al. Association between fear of falling and visuospatial and executive functions in older adults with subjective cognitive decline: a cross-sectional study. J Am Med Dir Assoc. 2025;26:105500. 39956154 10.1016/j.jamda.2025.105500

[23] St George RJ, Hinder MR, Puri R, Walker E, Callisaya ML. Functional near-infrared spectroscopy reveals the compensatory potential of pre-frontal cortical activity for standing balance in young and older adults. Neuroscience. 2021;452:208–18. 33197501 10.1016/j.neuroscience.2020.10.027

[24] Stern Y. Cognitive reserve. Neuropsychologia. 2009;47:2015–28. 19467352 10.1016/j.neuropsychologia.2009.03.004 PMC2739591

[25] Stojan R, Mack M, Bock O, Voelcker-Rehage C. Inefficient frontal and parietal brain activation during dual-task walking in a virtual environment in older adults. Neuroimage. 2023;273:120070. 37004827 10.1016/j.neuroimage.2023.120070

[26] Strangman G, Culver JP, Thompson JH, Boas DA. A quantitative comparison of simultaneous BOLD fMRI and NIRS recordings during functional brain activation. Neuroimage. 2002;17:719–31. 12377147

[27] Takakusaki K. Functional neuroanatomy for posture and gait control. J Mov Disord. 2017;10:1–17. 28122432 10.14802/jmd.16062 PMC5288669

[28] Takizawa R, Fukuda M, Kawasaki S, Kasai K, Mimura M, Pu S, et al. Neuroimaging-aided differential diagnosis of the depressive state. Neuroimage. 2014;85(Pt 1):498–507. 23764293 10.1016/j.neuroimage.2013.05.126

[29] Teo WP, Goodwill AM, Hendy AM, Muthalib M, Macpherson H. Sensory manipulation results in increased dorsolateral prefrontal cortex activation during static postural balance in sedentary older adults: an fNIRS study. Brain Behav. 2018;8:e01109. 30230687 10.1002/brb3.1109 PMC6192391

[30] Tinetti ME, Richman D, Powell L. Falls efficacy as a measure of fear of falling. J Gerontol. 1990;45:P239-243. 2229948 10.1093/geronj/45.6.p239

[31] von Lühmann A, Ortega-Martinez A, Boas DA, Yücel MA. Using the general linear model to improve performance in fNIRS single trial analysis and classification: a perspective. Front Hum Neurosci. 2020;14:30. 32132909 10.3389/fnhum.2020.00030 PMC7040364

[32] Whipple MO, Hamel AV, Talley KMC. Fear of falling among community-dwelling older adults: a scoping review to identify effective evidence-based interventions. Geriatr Nurs. 2018;39:170–7. 28941942 10.1016/j.gerinurse.2017.08.005 PMC5862787

[33] Xiong J, Xu X, Lu W, Wang P, He L, Wu Y. Global prevalence of fear of falling among older adults: a systematic review and meta-analysis. Arch Gerontol Geriatr. 2024;113:105043.

[34] Xu G, Zhou M, Chen Y, Song Q, Sun W, Wang J. Brain activation during standing balance control in dual-task paradigm and its correlation among older adults with mild cognitive impairment: a fNIRS study. BMC Geriatr. 2024;24:144. 38341561 10.1186/s12877-024-04772-1 PMC10859010

[35] Yücel MA, Lühmann AV, Scholkmann F, Gervain J, Dan I, Ayaz H, et al. Best practices for fNIRS publications. Neurophotonics. 2021;8:012101. 33442557 10.1117/1.NPh.8.1.012101 PMC7793571

[36] Zhao Y, Qiu L, Sun Y, Huang C, Li T. Optimal hemoglobin extinction coefficient data set for near-infrared spectroscopy. Biomed Opt Express. 2017;8:5151–9. 29188110 10.1364/BOE.8.005151 PMC5695960

